# Abnormalities in circadian blood pressure variability and endothelial function: pragmatic markers for adverse cardiometabolic profiles in asymptomatic obese adults

**DOI:** 10.1186/1475-2840-9-58

**Published:** 2010-09-24

**Authors:** Alok K Gupta, Germaine Cornelissen, Frank L Greenway, Vijay Dhoopati, Franz Halberg, William D Johnson

**Affiliations:** 1 Pennington Biomedical Research Center, Louisiana State University System, Baton Rouge, Louisiana, USA; 2 Amarika Family Medicine, Durham/Roxboro, North Carolina, USA; 3 University of Minnesota, Minneapolis, Minnesota, USA

## Abstract

**Background:**

Cardiovascular disease (CVD) risk, although perceived to be high, is often difficult to demonstrate in disease free (healthy) obese adults.

**Hypothesis:**

Changes in circadian blood pressure variability (CBPV) and endothelial function (EF) may be early correlates of cardiometabolic disorders.

**Methods:**

Asymptomatic men and women in 3 groups: normal weight (n = 10), overweight (n = 10) and obese (n = 15) were evaluated. Blood pressure and heart rate were recorded over 7 days: every 30 minutes during the day and every 60 minutes during the night, by automatic ambulatory monitoring. Resting EF was assessed in a fasting state between 8-10 AM by brachial ultrasound. Anthropometric and cardiometabolic indicators were measured and correlations with CBPV and EF were investigated.

**Results:**

The 3 groups had (Mean(SD)) BMI: 22.6(1.6), 27(3) and 34(5) kg/m^2^, respectively, weight: 64(16), 79(14), 95(16) kg and waist circumference: 79(9), 93(10), 107(13) cm. None in normal-weight or overweight groups had abnormal CBPV, while 8 of 15 obese adults had one or more CBPV abnormities (p < 0.05). Obese adults with CBPV abnormalities had elevated hs-CRP (15.3(9.3) mg/L), fibrinogen (593(97) mg/dl), fasting serum glucose (102(16) mg/dL), and cardiac risk ratios (Total-C/HDL-C: 5.2(1.9), LDL-C/HDL-C: 3.1(1.4)). Adults in the 3 respective groups who did not have CBPV abnormalities had flow-mediated brachial artery dilatation (FMD) of 0.22(0.06); 0.20(0.04), 0.23(0.02) mm over resting diameter. Obese participants with CBPV abnormalities (Mesor-hypotension, circadian hyper amplitude tension, elevated pulse pressure), had attenuated FMD at 78, 52, and 56% of resting reference diameter (means 0.18(0.07), 0.12(0.08), and 0.13(0.05) mm; p < 0.05), respectively.

**Conclusions:**

Asymptomatic obese adults with abnormal CBPV and EF exhibit unfavorable cardiometabolic profiles.

## Introduction

Obesity with its increasing prevalence, and as a consequence of its associated co-morbidities, is rapidly becoming the leading global cause for cardiovascular morbidity and mortality [1,2]. Cardiovascular disease (CVD) remains the number one cause of death, not only in the United States [3], but also worldwide [4]. The conventional risk factors: age, gender, smoking status, diabetes mellitus (DM), hypertension (HTN), dyslipidemia (DysL), and metabolic syndrome (MetS), are all known to have strong positive associations with the risk for CVD-related adverse events [5,6]. The obesity epidemic has, however, altered the paradigm for assessing CVD risk with factors like DM, HTN, DysL, and the MetS.

Diabetes mellitus, the well-recognized CVD risk equivalent [7,8], where obtaining tight glycemic control is thought to reduce the enhanced CVD risk [9], is exacerbated by the overweight or obese status. Due to an increasing recognition that CVD risk remains high when serum glucose concentrations are greater than 100 mg/dL [10], and that this enhanced CVD risk can be covertly present as far back as 15 years prior to the overt loss of glycemic control [11], asymptomatic (disease-free) overweight or obese adults with prediabetes (ADA criteria: an impaired fasting glucose (IFG) and/or impaired glucose tolerance (IGT)), could also have an increased risk of developing CVD [12]. Prediabetes is associated with early carotid atherosclerosis [13], coronary artery calcification [14], as well as other vascular abnormalities. Our own recent findings indicate that prediabetes is associated with abnormal circadian BP variability [15], and that exacerbated proinflammatory milieu in obese is associated with prediabetes and prehypertension [16].

Hypertension [17] and dyslipidemia [18]., similarly associated with increased CVD risk, are also intensified by the overweight and obese status. Most adults with HTN are overweight. The obese are six times more likely to have high blood pressure compared to those that are normal weight [17]. Asymptomatic overweight and obese with dysglycemia (prediabetes), dysregulation of blood pressure (prehypertension) and/or abnormal metabolic measures (premetabolic syndrome) are often unrecognized as having the metabolic syndrome [19]: a cluster of risk factors with underlying systemic inflammation, insulin resistance, and compensatory hyperinsulinemia [20]. Metabolic syndrome has been shown to be related to myocardial infarction (OR, 2.01; 95% CI, 1.53- 2.64), stroke (OR, 2.16; 95% CI, 1.48-3.16), and myocardial infarction/stroke (OR, 2.05; 95% CI, 1.64-2.57), in both women and men [21].

Early recognition of an elevated risk for developing CVD remains highly desirable as two thirds of unexpected cardiac deaths occur in adults without prior recognition of disease [22]. A third of the women placed at low risk with conventional risk assessment measures have significant subclinical atherosclerosis [23]. At age 40, the lifetime risk for coronary heart disease is 1 in 2 for men, 1 in 3 for women [24]; and for stroke, it is 1 in 6 for men, 1 in 5 for women [25]. With the alteration of conventional risk assessors due to an increasing body weight, and the largely unsubstantiated perceived increase in CVD risk in clinically healthy adults with altered weight, newer methods for risk recognition are clearly warranted.

Normal blood pressure is characterized by a circadian variation which includes generally higher day-time, and lower night-time pressures, a night time descent and an early morning surge. A spot blood pressure measured in a physician's office is an isolated cross-sectional view of this circadian pattern and varies depending on the time of the day it is obtained. Blood pressure automatically measured at fixed intervals for 7 days (with devices for ambulatory use) provides ample data for an unequivocal assessment of either normality or abnormality which is undetected by spot office BP measurements. Resting endothelial function obtained in a fasting state and during fixed clock hours, is reflective of the early perturbation caused by adverse cardiometabolic factors.

This study investigated the overall hypothesis that subtle changes in normal circadian BP variability and endothelial function are influenced by the progressive visceral adipose tissue expansion, the accompanying alteration of the metabolic milieu (glycemic and systemic pro-inflammatory changes) and the overall vascular response to these metabolic perturbations. Taken together, these subtle alterations in disease-free obese men and women could, over the long term, accelerate cardiovascular disease related adverse events.

## Materials and methods

### Study design

Disease-free (or otherwise healthy) obese men and women with no co-morbidities, screening for a weight loss study at the Outpatient Clinic, Pennington Biomedical Research Center (PBRC), were offered an opportunity to have 7-day automatic ambulatory blood pressure (BP) monitoring and endothelial function assessment. The Pennington Biomedical Research Center is a campus of the Louisiana State University System and conducts basic, clinical and population research. The research enterprise at the Center includes 80 faculty and more than 40 post-doctoral fellows who comprise a network of 57 laboratories. These are supported by lab technicians, nurses, dieticians, and support personnel, and 19 highly specialized core service facilities. The Center's nearly 600 employees occupy several buildings on a 234-acre campus.

Each candidate was provided with the PBRC Institutional Review Board approved informed consent and followed standard consenting procedures. Fifteen obese adults agreed to participate in the automatic ambulatory BP monitoring and endothelial function assessment. Demographic and anthropometric information along with a resting EKG were obtained, followed by a comprehensive medical history and a complete physical examination. The initial visit ended with teaching and demonstration of the use, of an automatic blood pressure monitor for ambulatory use. Upon completion of 7-day ambulatory BP monitoring, a fasting blood draw, and a resting endothelial function assessment were done. Comparison groups of 10 disease-free normal-weight, and 10 disease-free overweight adults were obtained from other clinical trials. They also consented for automatic ambulatory BP monitoring and endothelial function assessment and were processed in a similar fashion.

### Study population

Healthy (disease-free) normal-weight, overweight and obese (n = 35) non-smoking men and women between the ages of 30-75 years, with no personal history of or ongoing treatment for any chronic medical conditions.

### Inclusion/exclusion criteria

Apparently healthy men and women between the ages of 30-75 years, with no history of DM, HTN, DysL, MetS, and/or CVD were included. Adults with a personal history of or ongoing treatment for diabetes, hypertension or any other chronic cardiac, renal, gastro-intestinal, pulmonary or any other systemic disease process requiring chronic intake of prescription medications, were excluded.

### Seven-day automatic ambulatory blood pressure monitoring

An automatic BP monitoring device (Spacelabs^® ^Medical) for ambulatory use was attached to a BP cuff to obtain BP and heart rate (HR) readings at 30-min intervals during the day (6:30 AM to 9:30 PM) and 60-min intervals at night (10 PM to 6 AM) while the participants went about their activities. Data were downloaded into the database about mid-way and at the end of the 7-day recording span.

### Endothelial function by brachial ultrasound

Assessment of resting endothelial function was done in a fasting state, after having avoided stimulants for 12 hours, at the same fixed clock hour (range 8-10 AM), using a Toshiba^® ^brachial ultrasound device. This device uses 7.5 MHz multi-frequency linear array transducer and a MIA Vascular Tools Brachial Analyzer Version 5.8.1 to determine brachial artery diameter. This technique has been previously validated and is in use with our clinical core. After obtaining reference (resting) brachial artery diameter measures, a forearm BP cuff was used to occlude the brachial artery for 5 minutes. The increase in brachial artery diameter (over the reference measure) due to the flow-mediated dilatation of the brachial artery after the release of the occlusion, served as a measure of endothelial function. One subject from each group (normal-weight, over-weight and obese without circadian blood pressure variability abnormality) had a measure of endothelial function. One obese subject with each of the circadian variability abnormality (M-hypotension, CHAT and EPP) also had endothelial function assessment.

### Risks and discomforts

These non-invasive devices used a BP cuff that is inflated and released to obtain BP readings and data on endothelial function. The automatic ambulatory BP device attached to a BP cuff under the clothes was placed on the belt or carried in a pouch. Repeated measurements of BP and HR at timed intervals over 7 days, while the participant was at work or at home, allowed for acclimatization to the minimal discomfort, which is similar to having BP measured. Endothelial function was also obtained by using a BP cuff, coupled with an ultrasound probe that measures brachial artery diameter at rest (reference), during occlusion and the post-occlusion increase after release.

### Demographic, anthropometric and laboratory measures

Standard demographic and anthropometric measures were obtained for all the participants. Waist circumference (a surrogate marker for central adiposity), serum hs-CRP and fibrinogen (for the assessment of systemic inflammation), fasting serum glucose and HbA1C (for the assessment of glycemic status) and fasting complete lipid profile (for the assessment of serum lipid sub-fractions, and obtaining cardiac risk ratios) were obtained.

### Normal-weight, overweight, obese, normoglycemia, prediabetes, prehypertension and premetabolic syndrome

Participants were placed into normal weight (BMI < 25 kg/m^2^) overweight (BMI between 25-29.9 kg/m^2^) and obese (BMI > 30 kg/m^2^) categories. Normoglycemia and prediabetes, for the purpose of CVD risk assessment, were defined as a fasting serum glucose less than 100 mg/dL and a fasting serum glucose of more then 100 mg/dL but less then 126 mg/dL, respectively (impaired fasting glucose or IFG: ADA diagnostic criteria [26]). The diagnosis of prehypertension was based on resting (after a 5-minute rest) mean of (two successive assessments 1 minute apart) clinic BP measures of systolic (S) BP > 120 but < 139 and/or diastolic (D) BP > 80 but < 89 mm Hg (JNC 7 criteria [27]). Premetabolic syndrome was defined using the diagnostic criteria for the metabolic syndrome (NCEP ATP III criteria), with the substitution of prediabetes and prehypertension criteria for glucose and BP measures.

### Data analysis and statistical methods

**Figure 1:**
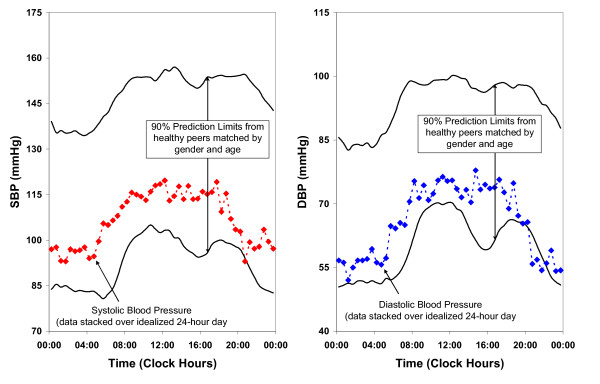
**Normal circadian blood pressure variability**.

ABPM data gathered at the PBRC were electronically sent to the Halberg Chronobiology Center, University of Minnesota for statistical analysis. Endothelial function data were analyzed at the PBRC and then merged with the ABPM data sent back from Dr. Halberg's laboratory. A summary in time (sphygmochron) was prepared that reported, among other measures, the midline-estimating-statistic of rhythm (MESOR or M), the timing of high values (acrophase), and the extent of predictable change within a day (double circadian amplitude) for SBP, DBP and HR. Normal circadian BP variability for SBP and DBP over a 24-hour span is shown in Figure 1. Seven-day monitoring ensures the consistency of either normality or abnormality over each one of the seven days and reports the average over the seven-day span.

**Figure 2:**
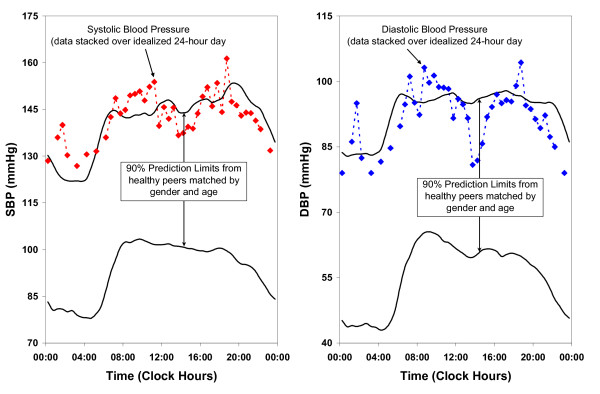
**MESOR-hypertension**.

MESOR Hypotension (M-Hypotension) or MESOR Hypertension (M-Hypertension) in this system is defined as a BP MESOR below the lower 5% or above the upper 95% prediction limit of peers matched by age and gender. M-Hypertension for SBP and DBP is shown in Figure 2.

**Figure 3:**
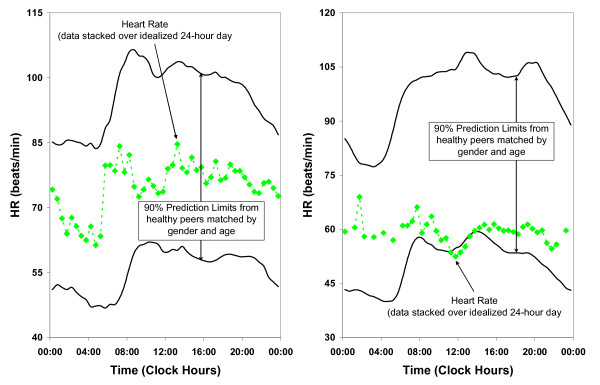
**Acceptable and decreased heart rate variability**.

**Figure 4:**
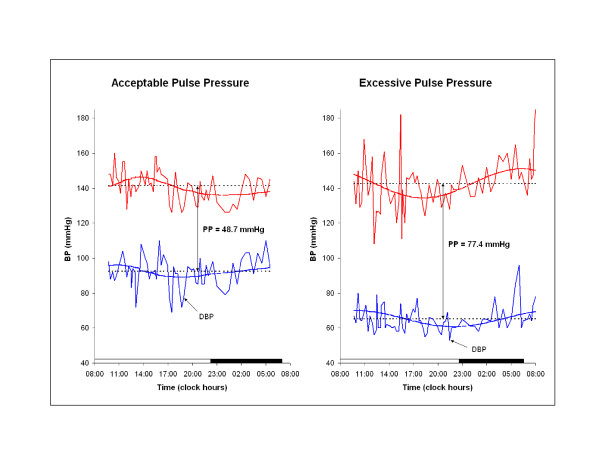
**Acceptable and elevated pulse pressure**.

Excessive circadian BP excursion (circadian hyper amplitude tension or CHAT) is a circadian double amplitude for SBP and/or DBP above the upper 95% prediction limit for peers. BP ecphasia is defined as an acrophase (timing) for BP (but not HR) occurring outside of the anticipated 90% prediction limits. HR variability is deficient (DHRV) when the standard deviation (SD) of HR is < 7.5 bpm. Pulse pressure is elevated (EPP) when it exceeds 60 mmHg. DHRV and EPP are shown in Figure 3 and Figure 4, respectively.

Day-night ratios were also computed for the 7-day record as a whole and for each day separately, for a classification in terms of "dipping". Due to the normal daytime BP being more than the nighttime BP, a negative day-night ratio was classified as "reverse dipping". A positive day-night ratio between 10% and 20% was classified as "dipping", whereas a ratio of less than 10% or greater than 20% was classified as "non-dipping" or "excessive dipping", respectively.

The occurrence of any of the above abnormal circadian patterns of BP and/or HR, overall and for each day separately, in terms of circadian characteristics and in terms of the day-night ratio, was determined for each individual in the three groups (normal-weight, overweight and obese). Serial measurement over 7 days ensured the consistency of either normality or abnormality. The groups were compared using an Exact Test or Poisson regression for frequency of occurrence data and Analysis of Variance of quantitative measurements.

## Results

Table 1 describes the clinical characteristics and details the circadian BP variability abnormalities and endothelial function in the normal-weight, overweight, and obese adults. The 3 groups, consisting of normal-weight (Mean ± SD; 52 ± 13 y, range 32-71 y), overweight (52 ± 7 y, range 40-62 y) and obese (56 ± 10 y, range 41-70 y) adults, were not different in age, but by design had an incremental increase in weight and BMI. All of the twenty normal-weight and overweight participants had normal circadian BP variability, while 8 out of 15 obese participants had at least one or more *variety *of *abnormal *circadian BP variability (four participants had one abnormality each, while the other four had two variability abnormalities each) (p < 0.05; Exact Test). The 8 obese participants had a total of twelve circadian BP variability abnormalities: systolic and/or diastolic MESOR-hypotension (M-Hypotension: 5/10; p < 0.05) or -hypertension (M-Hypertension: 2/10), excessive BP excursion (CHAT: 1/10) and/or elevated pulse pressure (EPP: 4/10; p < 0.05). Overall, of the total fifteen obese participants, 7 obese participants had normal circadian variability and 8 obese participants had twelve circadian BP variability abnormalities. No abnormalities were found with the day-night ratios.

**Table 1:** Clinical characteristics, circadian BP variability and endothelial function in disease free normal-weight, overweight and obese adults

	Normal (n = 10)	Overweight (n = 10)	Obese (n = 15)	P
**Age (Y)**	52 ± 12	52 ± 7	56 ± 10	NS *
**Gender (W/M)**	4/6	3/7	10/5	**NS**
**Weight (kg)**	**64 ± 16**	**79 ± 14**	**95 ± 16**	NA *
**BMI (kg/m^2^)**	**22.6 ± 3**	**27.3 ± 3**	**33.9 ± 5**	NA *
**Waist Circ (cm)**	79 ± 9	93 ± 10	**117 ± 13**	< 0.05 *
**Abnormal CBPV**	0/10	0/10	**8/15**	< 0.05 **
**MESOR-Hypotension**	0/10	0/10	**5/15**	< 0.05 **
**MESOR-Hypertension**	0/10	0/10	2/15	**NS**
**CHAT^1^**	0/10	0/10	1/15	**NS**
**EPP^2^**	0/10	0/10	**4/15**	< 0.05 **
**Total CBPV abnormalities**	0/10	0/10	**12/15**	< 0.05 **
**Endothelial Dysfunction**	0/1	0/1	**3/4**	< 0.05 **

^1^Circadian Hyper-Amplitude Tension

^2^Elevated Pulse Pressure

DATA: MEAN ± SD

* Analysis of Variance

**Exact test

Normal-weight and overweight participants had a flow-mediated brachial artery dilatation of 0.22 ± 0.06 and 0.20 ± 0.04 mm over resting (above reference) diameter, respectively. Obese participants without circadian BP abnormalities had a similar (to normal and overweight) flow-mediated brachial artery dilatation of 0.23 ± 0.02 mm over resting (above reference) diameter, compared to an attenuated dilation of 0.18 ± 0.07, 0.12 ± 0.08, and 0.13 ± 0.05 mm in those obese participants who had circadian BP variability abnormalities (M-Hypotension, CHAT and EPP, at 78, 52 and 56% of the expected; p = 0.3, 0.05 and 0.006, respectively).

Table 2 summarizes the cardiovascular disease risk profile in the normal-weight (n = 10) and overweight (n = 10) with normal CBPV and EF and the obese (n = 8) adult participants with abnormal CBPV. The normal-weight and overweight participants had a significantly lower mean waist circumference than the obese participants with both the women and the men well below the entry threshold of 88 and 102 cm, respectively, for inclusion in the metabolic syndrome. The obese participants, on the other hand with a statistically significantly higher waist circumference met the criteria for inclusion into the metabolic syndrome. The normal-weight and overweight adults had a normal spot office SBP/DBP, pulse pressure of 41 ± 7, 43 ± 8 mm Hg and heart rate of 67 ± 9, 74 ± 12 bpm. The 8 obese participants with abnormalities, however, had *prehypertension *(JNC 7 criteria: SBP 120-139 and/or DBP 80-80 mm Hg) with normal pulse pressure and heart rate. The normal-weight and overweight participants had normal fasting serum glucose, contrasted with the 8 obese participants with abnormalities who had *prediabetes *(ADA criteria: FSG 100-125 mg/dL). The normal-weight and overweight participants had normal lipid profiles with desirable total-C, triglycerides, HDL-C, and LDL-C along with desirable cardiac risk ratios. The 8 obese participants with abnormalities had greater than the desirable total-C, triglycerides, LDL-C and less than desirable HDL-C. Their cardiac risk ratios were also over the desirable range.

**Table 2:** CVD risk in disease free normal-weight and overweight subjects and in obese adults with CBPV abnormalities

	Normal (n = 10)	Overweight (n = 10)	Obese (n = 8)	P**
WC (Women < 88 cm)*	71 ± 4	84 ± 6	***109 ± 15***	**< 0.05**
WC (Men < 102 cm)*	84 ± 7	97 ± 9	***114 ± 6***	**< 0.05**
SBP (< 120 mm Hg)*	113 ± 8	118 ± 10	***129 ± 12***	**NS**
DBP (< 80 mm Hg)*	72 ± 5	75 ± 5	***80 ± 6***	**NS**
FSG (< 100 mg/dL)*	89 ± 5	89 ± 8	***102 ± 16***	**< 0.05**
Total-C (< 200 mg/dL)*	181 ± 23	180 ± 20	***223 ± 38***	**< 0.05**
LDL-C (< 130 mg/dL)*	109 ± 25	96 ± 13	***133 ± 34***	**< 0.05**
HDL-C (> 50 mg/dL)*	63 ± 4	54 ± 16	***46 ± 13***	**NS**
TG (< 150 mg/dL)*	49 ± 12	112 ± 60	***220 ± 111***	**< 0.05**
Total-C/HDL-C ratio (< 5)*	2.9 ± 0.6	3.6 ± 0.9	***5.2 ± 1.9***	**NS**
LDL-C/HDL-C ratio (< 3)*	1.7 ± 0.5	1.9 ± 0.7	***3.1 ± 1.4***	**< 0.05**
***Numbers *in *Italics *are *outside *the *desirable range***				

DATA: MEAN ± SD

* (In parenthesis: Desirable values)

** Analysis of Variance

Table 3 compares the seven obese participants with normal circadian BP variability with the eight obese participants who had abnormalities. The seven obese participants (BMI 32 kg/m^2^) who had normal circadian BP variability had normal glucose, hs-CRP, fibrinogen, triglycerides, HDL-C and cardiac risk ratios. In contrast the eight obese subjects with abnormal circadian BP variability exhibited majority of the CVD risk parameters outside of the desirable range.

**Table 3:** CVD risk in disease free obese adults without and with abnormal CBPV

	Obese with Normal CBPV (n = 7)	Obese with Abnormal CBPV (n = 8)
**BMI (kg/m^2^)**	*32 ± 5*	***36 ± 3***
WC (F < 88 cm)*	*95 ± 13*	***113 ± 13***
WC (M < 102 cm)*	*109 ± 13*	***111 ± 5***
SBP (< 120 mm Hg)*	*121 ± 12*	***129 ± 12***
DBP (< 80 mm Hg)*	*82 ± 5*	***80 ± 6***
FSG (< 100 mg/dL)*	94 ± 6	***102 ± 16***
hs-CRP(< 3.0 mg/L)*	1.9 ± 1.7	***15 ± 9***
Fibrinogen (< 450)*	411 ± 13	***593 ± 97***
HDL-C (> 50 mg/dL)*	52 ± 12	***46 ± 13***
TG (< 150 mg/dL)*	117 ± 47	**133 ± 35**
Total-C/HDL-C ratio (< 5)*	3.9 ± 0.7	***5.2 ± 1.9***
LDL/HDL-C ratio (< 3)*	2.4 ± 0.6	***3.1 ± 1.4***
***Numbers *in *Italics *are *outside *the *desirable range***		

DATA: MEAN ± SD

* (In parenthesis: Desirable values)

**Figure 5:**
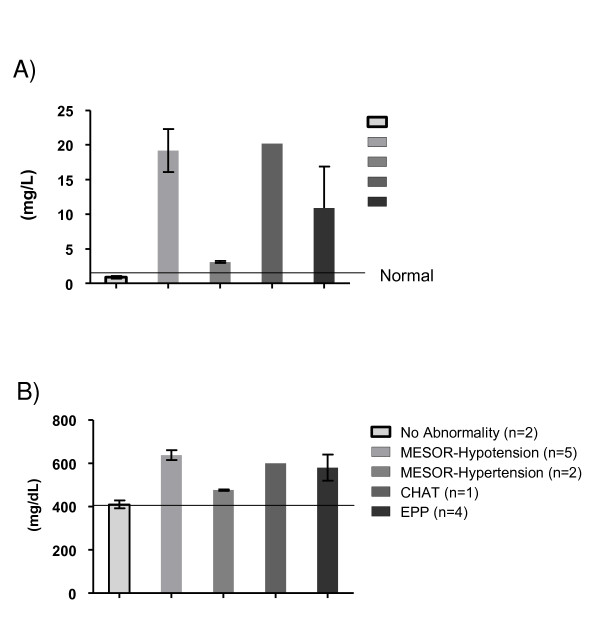
**Pro-inflammatory milieu in obese adults**. (A): hs-CRP: normal and abnormal CBPV. (B): fibrinogen: normal and abnormal CBPV.

Figure 5 details the pro-inflammatory milieu in the obese adults. Panel (5 A) depicts serum high sensitivity C-reactive protein (hs-CRP) concentrations in the obese adults with normal and abnormal circadian BP variability. Obese adults with no abnormalities (n = 7) had normal hs-CRP concentrations of (Mean ± SEM) 1.9 ± 1.7 mg/L. Participants with abnormal circadian BP variability disorders: M-Hypotension (n = 5) had hs-CRP concentration of 19.2 ± 3.1 mg/L (p < 0.05), with M-Hypertension (n = 2) 3.1 ± 0.1 mg/L (p < 0.05), with CHAT (n = 1) 20.2 mg/L and with EPP (n = 4) 10.9 ± 6 mg/L. Panel (5 B) depicts serum fibrinogen concentrations in the obese adults with normal and abnormal circadian BP variability. Obese adults with no abnormalities (n = 7) had normal fibrinogen concentrations (Mean ± SEM) of 411 ± 18 mg/dL. Participants with abnormal circadian BP variability disorders: M-Hypotension (n = 5) had fibrinogen concentration of 638 ± 38 mg/dL (p < 0.05), with M-Hypertension (n = 2) 477 ± 7 mg/dL, with CHAT (n = 1) 600 mg/dL and with EPP (n = 4) 581 ± 60 mg/dL.

**Figure 6:**
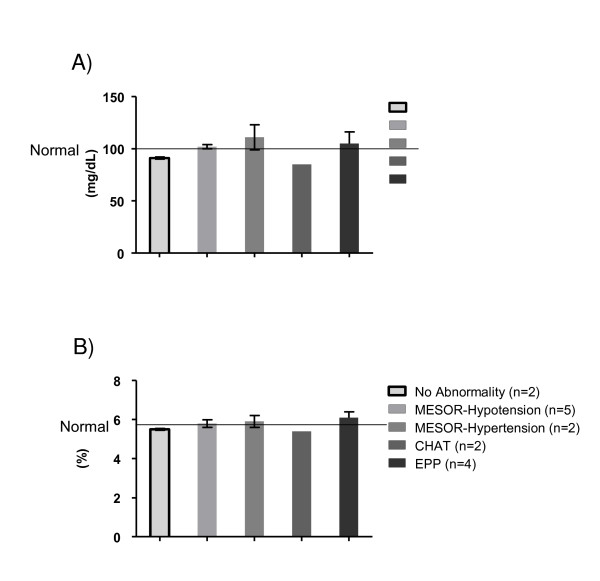
**Glycemic milieu in obese adults**. (A): FSG: normal and abnormal CBPV. (B): HbA1c: normal and abnormal CBPV.

Figure 6 details the glycemic milieu in the obese adults. Panel (6 A) depicts fasting serum glucose (FSG) concentrations in the obese adults with normal and abnormal circadian BP variability. Obese adults with no abnormalities (n = 7) had normal FSG concentrations (Mean ± SEM) of 94 ± 6 mg/dL. Participants with abnormal circadian BP variability disorders: M-Hypotension (n = 5) had FSG of 102 ± 2 mg/dL (p < 0.05), with M-Hypertension (n = 2) 111 ± 24 mg/dL, with CHAT (n = 1) 85 mg/dL and with EPP (n = 4) 105 ± 11 mg/L. Panel (6 B) depicts percent glycosylated hemoglobin (HbA1C) in the obese adults with normal and abnormal circadian BP variability. Obese adults with no abnormalities (n = 7) had normal HbA1C (Mean ± SEM) of 5.5 ± 0.05%. Participants with abnormal circadian BP variability: M-Hypotension (n = 5) had HbA1C of 5.8 ± 0.3% (p < 0.05), with M-Hypertension (n = 2) 5.9 ± 0.6%, with CHAT (n = 1) 5.4% and with EPP (n = 4) 6.1 ± 0.3%.

**Figure 7:**
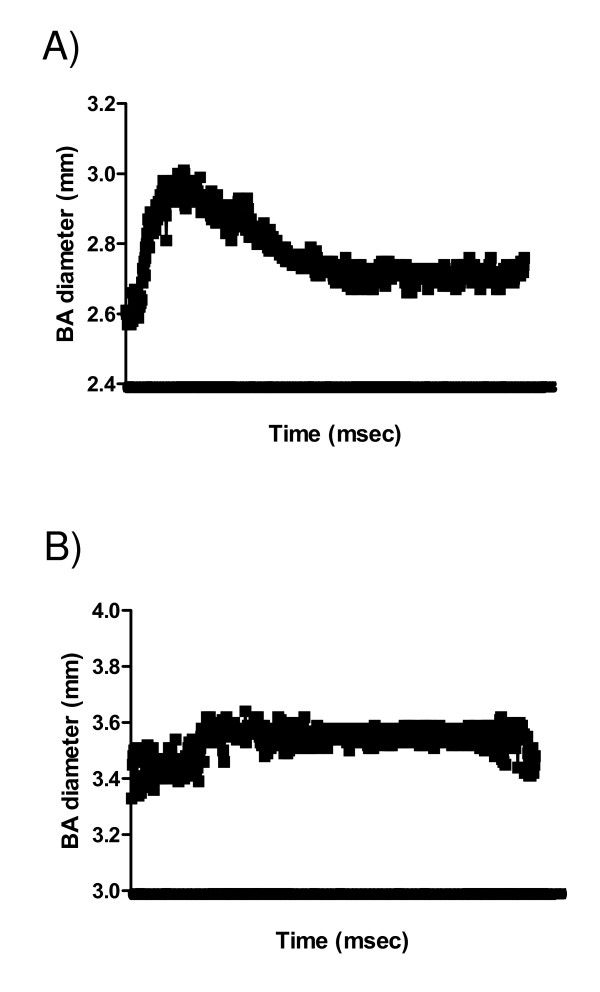
**Endothelial function in obese adults**. (A): Normal CBPV. (A): Abnormal CBPV.

Figure 7 illustrates the normal and abnormal flow-mediated brachial artery dilation curves in a representative obese adult with and without circadian BP variability abnormalities. Brachial artery dilation upon release of occlusion above the resting (reference) measure reported in millimeters is the measure of endothelial function. Panel (7 A) shows an increase in brachial artery diameter after release of brachial artery occlusion, representing normal endothelial function in an obese adult with no circadian BP variability abnormalities. Panel (7 B) shows a flatter brachial artery diameter after release of brachial artery occlusion, representing endothelial dysfunction in an obese adult with circadian BP variability abnormalities.

## Discussion

The data from this study show that only those asymptomatic (disease free) obese adults (that had prediabetes, prehypertension, elevated systemic inflammation and cardiac risk ratios), when compared with disease-free normal-weight, overweight and obese adults (with normoglycemia, desirable blood pressure, systemic inflammation and cardiac ratios) have significant circadian BP variability abnormalities and endothelial dysfunction. Circadian variability of blood pressure and heart rate measured over 7 days provides a dynamic, functional assessment of the cardiovascular system. It also ensures the consistency of either normality, or abnormality of the circadian BP variability measures. Both the obese participants with M-Hypertension also had an elevated pulse pressure. Of the five obese participants with M-Hypotension, two also had an elevated pulse pressure. Thus four of the eight participants with abnormal BP variability had, not one, but two varieties of circadian BP variability abnormalities (8 obese participants had 12 variability abnormalities). Normal-weight, overweight and obese participants had a flow-mediated brachial artery dilatation of 0.22 ± 0.06, 0.20 ± 0.04 and mm over resting (above reference) diameter, compared to an attenuated dilation of 0.18 ± 0.07, 0.12 ± 0.08, and 0.13 ± 0.05 mm in those obese participants who had circadian BP variability abnormalities.

### Abnormal circadian BP variability, endothelial function and cardiovascular morbidity and mortality

The effect of abnormal circadian BP variability upon untoward cardiovascular events is both separate and additive. Having more than one abnormality increases this risk: In a reference population of 214 patients (some with M-Hypertension) presenting with none of the 3 abnormal variability measures (decreased heart rate variability (DHRV), EPP, or CHAT), morbidity within a 6-year follow-up was found in 8 cases (3.7%). The presence of one abnormality (DRHV or EPP) alone raised the incidence of morbidity to 30.8%. When these two risk factors (DRHV and EPP) were both present, morbidity was doubled (66.7%). The presence of CHAT increased morbidity from 3.7% to 23.5% in the absence of the other two risk factors, from 30.8% to 50% when either DHRV or EPP was also present, or from 66.7% to 100% when all 3 risk factors are present [28]. Halberg et al. [29] have shown that even in the absence of conventional CVD risk factors (like DM, HTN, and DysL); abnormalities in circadian BP variability are risk factors for CVD and early death. Others have shown the superior predictive ability of ambulatory BP monitoring data vs. conventional office BP measures in adults with hypertension.

The endothelium is a highly active organ which regulates intravascular homeostasis by integrating numerous functions such as glycemia, blood pressure, pro-inflammatory/anti-inflammatory processes and coagulation. Endothelial dysfunction, the initial perturbation in the process of atherosclerosis, in asymptomatic individuals portends increased vascular disease risk [30]. Obese adults with abnormal circadian BP variability (M-Hypotension, CHAT, and EPP) also had endothelial dysfunction (flow mediated brachial diameter increase of 0.18(0.07), 0.12(0.08), and 0.13(0.05) mm over resting diameter or flow mediated dilation at only 78, 52, and 56% of the resting diameter, respectively, p <0.05). Abnormalities of both these measures: abnormal circadian BP variability (reflective of the functional aspects of the cardiovascular system), and endothelial dysfunction (reflective of a sum total of various effectors including glycemia, blood pressure, coagulation, pro-inflammatory and anti-inflammatory factors) in asymptomatic (disease free) obese adults are novel non-invasive methods for assessing the dynamic aspects of cardiovascular disease risk.

### Recognized CVD risk factors

Gradual weight gain in clinically healthy overweight and obese adults is preferentially manifest as an enlarging waist circumference [31]. Abdominal obesity, clinically measured as an increased waist circumference and suggestive of an expanding visceral adipose tissue compartment, has an independent association with coronary heart disease [32]. Visceral adipose tissue, which strongly correlates with most metabolic risk factors [33], upon expansion, alters its usual and customary adipokine secretion menu [34]. There is an increased flux in the factors influencing the pro-inflammatory and renin-angiotensin-aldosterone system, in parallel with attenuation in the anti-inflammatory factors. The altered pro-inflammatory (increased) and anti-inflammatory (decreased) balance, among other reasons promotes insulin resistance [35]. Elevated HbA1c is related to new onset CVD over a relatively short follow-up period in both men and women without diabetes, who do not develop diabetes, even after adjustment for other major risk factors [36]. Dysregulated pro-inflammatory: anti-inflammatory balance, increased serum hs-CRP [37], total leukocyte count [38], serum uric acid [39], and decreased adiponectin [34] are associated with increased cardiovascular disease. Disease-free obese with an exacerbated proinflammatory milieu exhibit prediabetes and prehypertension [16]. Prediabetes is associated with abnormal circadian BP variability [15], prehypertension clusters with other CVD risk factors [40] and metabolic syndrome more strongly predicts CVD than its individual components [41]. An imbalance between the central and the peripheral clock mechanisms has recently been suggested as the cause for the endothelial function [42]. 24-hour ambulatory blood pressure measures have been used to predict target-organ disease and clinical outcome in patients with hypertension [43] and more recently, elevations in nocturnal BP have been shown to precede diabetic nephropathy in hypertensive patients with T2DM [44].

Overweight or obese adults with larger than normal waistline, along with subtle metabolic alterations, either with elevated FSG (prediabetes [26], elevated SBP and/or DBP (prehypertension [27]), and/or a combination of risk factors (premetabolic syndrome) are commonplace in routine clinical practice. These adults with unrecognized elevated CVD risk, more often than not, are lost to regular follow-up. This results in a lost opportunity for primary prevention of CVD. We believe that increased CVD in asymptomatic normal-weight, overweight and/or obese adults can readily recognized by the abnormal circadian BP variability, and endothelial dysfunction. Non-pharmacologic, as well as pharmacologic measures can be utilized to reverse these early abnormalities. Non-pharmacologic measures: a 7% weight loss from reference (improving glycemia), and increasing physical activity up to 150 minutes per week, (improving both glycemia and blood pressure) could be advocated. Pharmacologic measures: treatment with thiazolidinediones (reducing insulin resistance and pro-inflammatory milieu, along with remodeling of the adipose tissue), biguanides (reducing insulin resistance), angiotensin converting enzyme inhibitors or angiotensin receptor blockers (reducing blood pressure, improving glycemia and systemic inflammation) and HMG-CoA reductase inhibitors or statins (systemic inflammation, improving cardiac risk ratios and endothelial function) could be utilized.

The results from this study show that latent CVD risk in disease-free (healthy) obese adults assessed with no or low risk by conventional risk assessment methods, can be unmasked by simple non-invasive measures. The obese participants exhibiting normal circadian BP variability had normal endothelial function, normotension, normoglycemia and were within the desirable limits for systemic inflammation, triglycerides, HDL-C, and cardiac risk ratios. Asymptomatic obese participants with abnormal circadian BP variability and endothelial dysfunction also had: an increased visceral adipose tissue, a heightened pro-inflammatory profile, prediabetes, prehypertension and abnormal cardiac risk ratios. Abnormal circadian BP variability and endothelial dysfunction, taken together with the altered adverse cardiometabolic profile, are indicative of an unrecognized CVD risk in disease free obese men and women.

### Study limitations

The study has several limitations that warrant discussion. The study participants were adult asymptomatic volunteers who were screening for various clinical studies at the Pennington Center, and may not be representative of the general population. Further, it is a cross-sectional study in which the temporal sequence of emergence of dysregulated assessments is unknown. Finally, the small sample size may have compromised the power to detect population differences among the normal-weight, overweight, and obese groups. Despite these shortcomings, this investigation documents statistically significant novel findings of a clinical correlation between circadian BP variability and endothelial function abnormalities and systemic proinflammation, prediabetes, prehypertension, elevated cardiac risk ratios, and establishes a foundation for further investigation of the underlying mechanisms.

## Conclusion

While studies with larger numbers of participants are clearly indicated, these findings taken in conjunction with the recognized subtle abnormal circadian BP variability in prediabetes [15] strengthen our overall hypothesis that progressive visceral adipose tissue expansion with the accompanying systemic pro-inflammatory and glycemic changes [16] and the overall vascular response to these metabolic perturbations, influence circadian BP variability and endothelial function. Taken together with other anthropometric and laboratory measures, these are indicative of an enhanced CVD risk. Circadian BP variability and endothelial function, along with subtle abnormalities of pro-inflammatory and glycemic milieu, can be novel measures for recognizing latent CVD risk in otherwise asymptomatic obese and possibly in other populations.

## Competing interests

The authors declare that they have no competing interests.

## Authors' contributions

AKG conceived of the study and drafted the manuscript. GC performed the chronobiological analyses for the circadian blood pressure data. WTJ performed the statistical analysis. AKG, WTJ, VD, FLG, and FH edited the manuscript. All authors have read and approved the final manuscript.
